# FgRad50 Regulates Fungal Development, Pathogenicity, Cell Wall Integrity and the DNA Damage Response in *Fusarium graminearum*

**DOI:** 10.3389/fmicb.2019.02970

**Published:** 2020-01-09

**Authors:** Chengqi Zhang, Xuexiang Ren, Xintong Wang, Qiong Wan, Kejian Ding, Li Chen

**Affiliations:** ^1^Key Laboratory of Biology and Sustainable Management of Plant Diseases and Pests of Anhui Higher Education Institutes, College of Plant Protection, Anhui Agricultural University, Hefei, China; ^2^Institute of Plant Protection and Agro-Products Safety, Anhui Academy of Agricultural Sciences, Hefei, China

**Keywords:** *Fusarium graminearum*, FgRad50, fungal growth and virulence, deoxynivalenol, cell wall integrity, DNA damage response

## Abstract

Rad50 is a member of the double strand break repair epistasis group of proteins that play important roles in regulating DNA damage checkpoint signaling, telomere maintenance, homologous recombination and non-homologous end-joining in eukaryotes. However, the function of Rad50 in fungal plant pathogens remains unknown. In this study, we report the functional investigation of FgRad50 in the wheat head blight pathogen *Fusarium graminearum*. FgRad50 is an ortholog of *Saccharomyces cerevisiae* Rad50 that could restore the sensitivity of the yeast Rad50 mutant to DNA damage agents. The *FgRad50* deletion mutant (ΔFgRad50) exhibited defective vegetative growth, asexual/sexual development and virulence, as well as disrupted deoxynivalenol biosynthesis. Moreover, deletion of *FgRad50* resulted in hypersensitivity to DNA damage agents. Unexpectedly, FgRad50 plays a key role in responses to cell wall-damaging agents by negatively regulating phosphorylation of FgMgv1, a MAP kinase in the cell wall integrity (CWI) pathway. Taken together, these results suggest that FgRad50 plays critical roles in fungal development, virulence and secondary metabolism in *F. graminearum*, as well as CWI and the DNA damage response.

## Introduction

Fusarium head blight (FHB) is a destructive disease of wheat and barley caused by *Fusarium graminearum* species complex that occurs worldwide. This pathogen can lead to severe yield losses and produces mycotoxins such as deoxynivalenol (DON) and zearalenones (ZEN) that are harmful to human and animal health (Pestka and Smolinski, 2005; Kimura et al., 2007). FHB is difficult to control due to the lack of effective resistant wheat cultivars. Currently, the main strategy for controlling FHB remains the application of chemical fungicides, such as carbendazim, tebuconazole, and phenamacril (JS399-19) which has been marketed for controlling FHB in China, Li et al. (2008), McMullen et al. (2012). Previous studies showed that C14α-methylases encoded by *CYP51* genes are the targets of tebuconazole (Becher et al., 2011; Liu et al., 2011). However, carbendazim and phenamacril targets beta-tubulin and myosin I (FgMyo1), respectively, in *F. graminearum* (Zhang et al., 2015; Tang et al., 2018). Therefore, exploring potential targets is a priority for effective management of FHB.

DNA double-strand breaks (DSBs) arise from a variety of sources including exposure to radiation or carcinogens, and they can also spontaneously arise during DNA replication, resulting in DNA damage (Krejci et al., 2003). DSBs can be repaired by non-homologous end-joining (NHEJ) and homologous recombination (HR). NHEJ acts by directly ligating DNA ends with minimal or no base pairing at the junction (Mehta and Haber, 2014; Durdíková and Chovanec, 2017). By contrast, HR uses intact homologous duplex DNA sequences as templates for accurate repair (Daley et al., 2014; Mehta and Haber, 2014). Many components of these two DNA repair pathways have been identified. Although HR and NHEJ involve different sets of protein factors, Rad50, a component of the evolutionarily conserved MRX/MRN complex (Mre11-Rad50-Xrs2 in yeast or MRE11-Rad50-NBS1 in mammals), plays important roles in both pathways (Marsella et al., 2019). The MRX/MRN complex functions together with the Sae2 protein (CtIP in mammals) in initiation of DSB resection (Gobbini et al., 2016).

Rad50 belongs to the structural maintenance of chromosomes family and contains Walker-type nucleotide-binding motifs and a long coiled-coil domain (de Jager et al., 2001; Hopfner et al., 2002). Rad50 is an ATPase component of the MRX complex that generates single strands at DSB sites (Borde, 2007). Hydrolytic activity of Rad50 is crucial in the regulation of DNA binding, and hence all functions of the MRX complex (Deshpande et al., 2014).

The functions of Rad50 have been widely studied both in human and *Saccharomyces cerevisiae.* However, little is known about the roles of Rad50 in filamentous fungi. In the present study, we investigated the functions of Rad50 in *F. graminearum* to explore its potential exploitation as a drug target for the design of new antifungal agents against FHB. We found that FgRad50 is critical for hyphal growth, asexual and sexual development, DON production, pathogenicity, and DNA damage responses in *F. graminearum*.

## Materials and Methods

### Strains and Culture Conditions

*Fusarium graminearum* strain PH-1 (NRRL 31084) was used as a WT strain in this study. WT and mutant strains were grown on potato dextrose agar (PDA), minimal medium (MM), and complete medium (CM) at 25°C for mycelial growth tests (Correll et al., 1987). Strains were cultured in carboxymethyl cellulose (CMC) liquid medium for sporulation analysis (Harris, 2005). All strains were preserved as mycelia and conidia in 15% glycerol at −80°C.

### Yeast Complementation Assays

The full-length cDNA of *FgRad50* was amplified using primer pairs listed in Supplementary Table S1. The PCR product was cloned into the pYES2 vector (Invitrogen Co., CA, United States) using a One-Step Cloning Kit (Vazyme Biotech, Nanjing, China), and the resulting vector was transformed into the yeast *Rad50* mutant BY4741ΔRad50. Additionally, the empty pYES2 vector was transformed into WT BY4741 and mutant strains as controls. For complementation assays, yeast transformants were grown at 30°C on YPRG medium (1% yeast extract, 2% galactose, 2% bactopeptone) supplemented with 15 μg/mL camptothecin (CPT). All experiments were repeated three times independently.

### Construction of *FgRad50* Deletion Mutants and Complemented Strains

The ΔFgRad50 mutants were generated using the double-joint (DJ) PCR approach (Yu et al., 2004) and polyethyleneglycol (PEG)-mediated protoplast transformation (Proctor et al., 1995). Primers used to amplify the flanking sequences of the gene are listed in Supplementary Table S1. The open reading frame (ORF) of *FgRad50* was replaced with a hygromycin (*hyg*) resistance cassette. In order to confirm that the phenotype of *FgRad50* is caused by disruption of the gene, the DNA fragment carrying the native promoter and the ORF of *FgRad50* was amplified with the primer pair A7 + A8 (Supplementary Table S1). The resulting PCR products were cotransformed with *Xho*-digested *pYF11-GFP-Gen* vector into yeast cells XK1-25 using a yeast transformation kit (MP Biomedicals, Solon, OH, United States) to generate the recombined *pYF11-FgRad50-GFP-Gen* plasmid (Bruno et al., 2004; Chen et al., 2018). Then the *pYF11-FgRad50-GFP-Gen* plasmid was extracted from the yeast XK1-25 transformant using a yeast plasmid extract kit (Solarbio, Beijing, China) and then transferred into *Escherichia coli* strain DH5α to propagate the plasmids. Subsequently, the recombinant plasmid was transformed into the deletion mutant ΔFgRad50 to generate the complemented strains. For selective growth of complemented transformants, PDA medium supplemented with G418 sulfate (100 mg/L) was used. Deletion candidates and the complemented strain ΔFgRad50-C were identified by PCR assays with relevant primers and Southern blotting analyses (Supplementary Figure S1). The probe for Southern blotting analyses were labeled with digoxigenin using the high prime DNA labeling and detection starter kit II according to the manufacturer’s protocol (Roche Diagnostics, Mannheim, Germany).

### Sexual Reproduction and Growth Assays

For sexual reproduction analysis, aerial hyphae of 4-day-old cultures of each strain grown on carrot agar plates were placed in 500 μL of 2.5% Tween-60 solution, then perithecium formation and cirrhi production were assayed after incubation under black light at 25°C for 2 weeks (Nicholson, 2007). To determine the sensitivity to cell wall stress agents sodium dodecyl sulphate (SDS), congo red (CR), and calcofluor white (CFW), and to different DNA-damaging agents CPT, methyl methanesulfonate (MMS), hydroxyurea (HU) and 4-nitroquinoline (4-NQ), a mycelial growth assay was performed as described previously (Chen et al., 2019). The concentration of each compound is indicated in the figure legends. All experiments were repeated three times.

### Pathogenicity and DON Production Assays

Conidia of WT PH-1, ΔFgRad50 mutant, and ΔFgRad50-C complemented strains were harvested from cells grown in 4-day-old CMC medium by centrifugation and adjusted to a final concentration of 1 × 10^5^ conidia/mL in sterile distilled water. The pathogenicity assay was performed on wheat heads as previously described (Jiang et al., 2011). Briefly, a 10 μL aliquot of conidial suspension was injected into a floret in the central section spikelet of a single flowering wheat head of susceptible variety Annong8455. We included 15 replicates for each strain, infected spikelets in each inoculated wheat head were photographed 15 days after inoculation, and all experiments were repeated four times. To quantify DON production, infected spikelets of each strain were collected from inoculated wheat heads in the field, and total ergosterol was extracted and quantified from the infected spikelets as described previously (Liu et al., 2013). DON was then extracted, purified, and quantified using the liquid chromatography-tandem mass spectrometry (LC-MS/MS) method (Ji et al., 2014; Dong et al., 2016).

### qRT-PCR Assays

Fresh mycelia were harvested, ground in liquid nitrogen, total RNA was extracted using RNAiso Reagent (TaKaRa Co., Dalian, China), and 10 mg of each RNA sample was subjected reverse-transcribed using a HiScriptII Q RTSuperMix qPCR Kit (Vazyme Biotech, Nanjing, China). The qRT-PCR amplifications were performed in a CFX96^TM^ Touch (Bio-Rad, United States) using the SYBR Green I fluorescent dye detection. The expression level of each gene was determined by qRT-PCR with primers listed in Supplementary Table S1 and the expression of the *actin* gene was used as a reference. The experiment was repeated three times. The relative expression levels of genes in the wild-type strain and deletion mutant were calculated using the 2^–Δ^ ^Δ^ ^Ct^ method (Livak and Schmittgen, 2001). All experiments were repeated three times independently.

### Western Blotting Assays and Microscopy Imaging

Protein extraction and western blotting hybridization were performed as described previously (Yu et al., 2014). To measure the FgMgv1 phosphorylation level, WT, ΔFgRad50 and ΔFgRad50-C strains were inoculated into 200 mL liquid CM medium, cultured in a shaker (200 rpm) at 25°C for 36 h, mycelia were harvested, and total Mgv1 and phosphorylated Mgv1 were detected using an Mpk1 antibody (cat. Sc-6802; Santa Cruz, CA, United States) and an anti-phospho-p44/42 MAPK antibody (cat. 9101, Cell Signaling Technology), respectively. To further verify the FgRad50 expression after treatment with HU, immunoblot assay was performed using an anti-GFP antibody (cat. ab6556; Abcam, Cambridge, MA, United States). Anti-GAPDH antibody (Huaan Biotechnology, Hangzhou, China) was used as a reference. The experiment was conducted three times independently. The fluorescence intensity and localization of FgRad5-GFP tagged proteins were observed using a Zeiss LSM780 confocal microscope (Carl Zeiss AG, Jena, Germany).

### Statistical Analyses

Data are presented as the mean of triplicates ± standard deviations, and statistical significance was determined by Fisher’s least significant difference (LSD) test at *P* = 0.05.

## Results

### Identification of the *F. graminearum* Rad50 Ortholog

**FIGURE 1:**
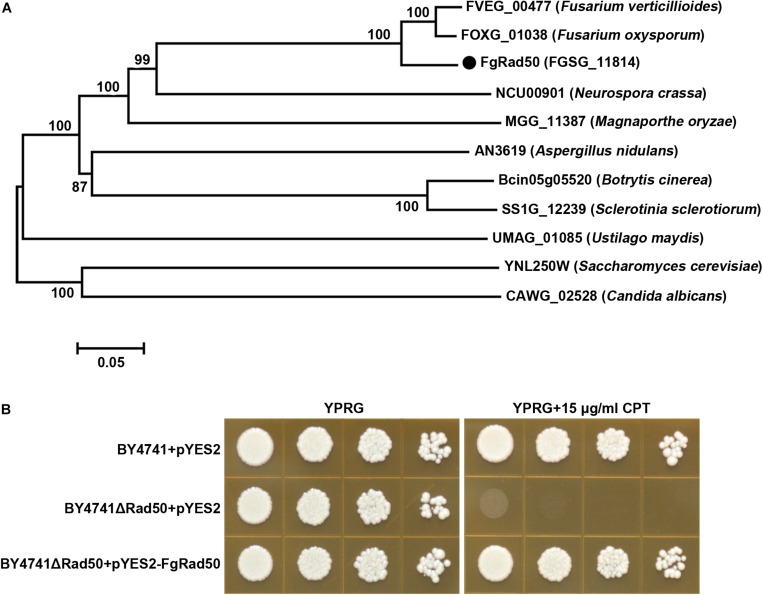
*Fusarium graminearum* FgRad50 encodes a functional homolog of *S. cerevisiae* Rad50. **(A)** Phylogenetic analysis of FgRad50 with other orthologs from other fungal species. The phylogenetic tree was constructed using MEGA 5.0 with full-length protein sequences and the neighbor-joining method with 1000 bootstrap replications. Amino acid sequences are from *F. graminearum (FGSG_11814*), *F. verticillioides* (*FVEG_00477*), *F. oxysporum* (*FOXG_01038*), *N. crassa* (*NCU00901*), *M. oryzae* (*MGG_11387*), *A. nidulans* (*AN3619*), *B. cinerea* (*Bcin05g05520*), *S. sclerotiorum* (*SS1G_12239*), *Ustilago maydis* (*UMAG_01085*), *C. albicans* (*CAWG_02528*), and *S. cerevisia*e (YNL250W). **(B)** FgRad50 restored the sensitivity of the yeast Rad50 mutant BY4741ΔRad50 to the DNA-damaging agent camptothecin (CPT). The Rad50 yeast mutant was complemented with FgRad50 to generate strain BY4741ΔRad50 + pYES2-FgRad50, which was spotted onto YPRG medium supplemented with 15 μg/mL CPT. In addition, wild-type (WT) BY4741 and mutant BY4741ΔRad50 strains were transformed with empty pYES2 vector and used as controls.

A homology search of the genome of *F. graminearum* PH-1 by BLASTp using the Rad50/YNL250W protein sequence of *S*. *cerevisiae* as the query revealed a high level of sequence identity between locus FGSG_11814 (Broad accession number, hereafter named FgRad50) and Rad50/YNL250W (*E*-value = 1.2e-142, sequence identity = 68%). To obtain more insight into Rad50 evolution in fungi, we retrieved genes encoding putative Rad50 proteins from another 10 fungal genomes available in FungiDB^1^. The protein sequences were aligned and phylogenetic analysis demonstrated that Rad50 is conserved among these fungal species (Figure 1A).

Analysis of the ΔRad50 *S. cerevisiae* mutant indicated a key role in eukaryotic DNA damage responses (Park et al., 2017). To characterize the functions of *FgRad50*, we first tested whether this gene could reverse defects in the ΔRad50 yeast mutant. As shown in Figure 1B, the growth of deletion mutant BY4741ΔRad50 was dramatically inhibited by DNA damage agent camptothecin (CPT). Additionally, the sensitivity of BY4741ΔRad50 to CPT was restored when expression vector pYES2 containing the full-length *FgRad50* cDNA was transformed into BY4741ΔRad50. These results indicate that FgRad50 and yeast Rad50 share a conserved function associated with DNA damage responses. However, multiple sequence alignments illustrated that a number of divergences in amino acid sequences between the FgRad50 and Rad50 orthologs in wheat and human (Supplementary Figure S2) and FgRad50 shares homology with *Homo sapiens* HsRad50 (identities 26.98%) and *Triticum turgidum* TtRad50 (identities 28.03%).

### Growth and Conidiation Are Defective in the ΔFgRad50 Mutant

**FIGURE 2:**
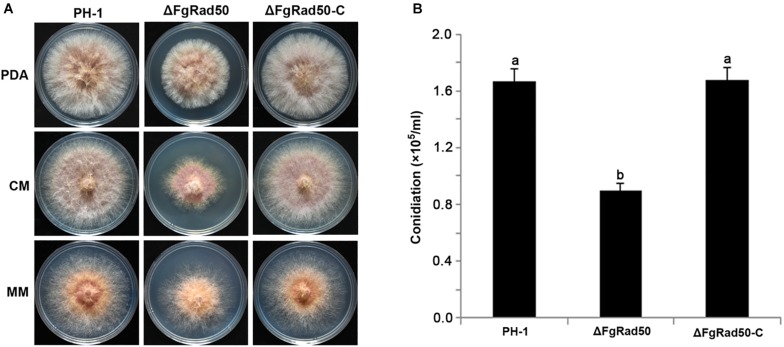
Effect of FgRad50 deletion on mycelial growth and conidiation in *F. graminearum.* **(A)** WT PH-1, deletion mutant ΔFgRad50, and ΔFgRad50-C complemented strains were grown on potato dextrose agar (PDA), complete medium (CM), and minimal medium (MM) at 25°C for 3 days. **(B)** Conidia were quantified after incubation of WT PH-1, deletion mutant ΔFgRad50, and complemented ΔFgRad50-C strains in 30 mL of carboxymethyl cellulose (CMC) liquid medium at 25°C with shaking at 180 rpm for 4 days. Bars denote standard deviations from three repeated experiments. The same letter on the bars for each treatment represents no significant difference at *P* = 0.05.

To investigate the roles of *FgRad50* in vegetative growth, the *FgRad50* deletion mutant ΔFgRad50, wild-type (WT) PH-1 cells, and the complemented ΔFgRad50-C strain were cultured on PDA, CM and MM agar plates. As shown in Figure 2A, the mycelial growth rate of ΔFgRad50 was slower than that of WT PH-1. Additionally, conidial production of each strain was evaluated by culturing in CMC conidia induction medium, and the results indicated that ΔFgRad50 produced significantly fewer conidia than WT PH-1 cells (Figure 2B). The phenotypic defects of the ΔFgRad50 mutant were restored by genetic complementation with WT *FgRad50* in the complemented strain ΔFgRad50-C. These observations indicate that *FgRad50* is required for vegetative growth and conidiation in *F. graminearum*.

### *FgRad50* Is Important for Sexual Reproduction

**FIGURE 3:**
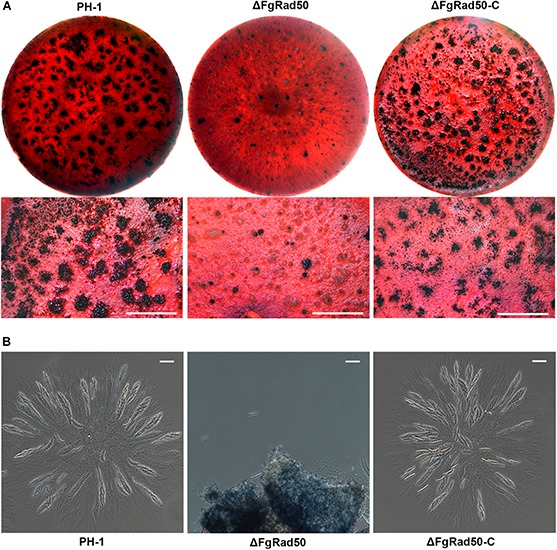
Analysis of defective sexual reproduction in the ΔFgRad50 mutant. **(A)** Perithecium formation in mating cultures of PH-1, ΔFgRad50 and ΔFgRad50-C examined at 3 weeks post-induction. Bar = 1 mm. **(B)** Dissection of perithecia showing asci and ascospores of WT PH-1, ΔFgRad50 and ΔFgRad50-C. Scale bar = 20 μm.

Since ascospores play a critical role in the infection cycle of *F. graminearum*, we also investigated sexual reproduction in PH-1, ΔFgRad50 and ΔFgRad50-C strains grown on carrot agar plates. At 15 days post-fertilization, WT and complemented strains produced mature perithecia with ascospore cirrhi (Figures 3A,B). Under the same conditions, the ΔFgRad50 mutant produced far fewer perithecia, and hardly any protoperithecia (Figures 3A,B), suggesting that *FgRad50* plays a key role in sexual reproduction in *F. graminearum*.

### Mutation of FgRad50 Alters Cell Wall Integrity

**FIGURE 4:**
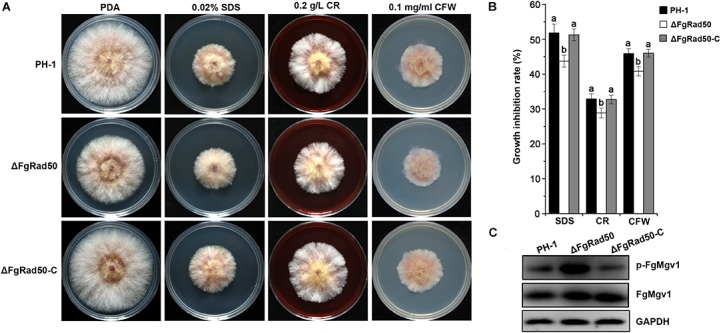
Role of FgRad50 in sensitivity to cell wall-damaging agents. **(A)** PH-1, ΔFgRad50 and ΔFgRad50-C were grown on PDA and PDA supplemented with 0.02% sodium dodecylsulphate (SDS), 0.2 g/L Congo Red (CR) or 0.1 mg/mL Calcofluor White (CFW). All plates were incubated at 25°C for 3 days. **(B)** Mycelial growth inhibition rates of PH-1 and mutants on various media. Bars denote standard errors from three experiments. Values above bars followed by the same letter indicate no significant difference at *P* = 0.05. **(C)** Comparison of phosphorylation levels of FgMgv1 in PH-1, ΔFgRad50 and ΔFgRad50-C. Phosphorylated FgMgv1 (p-FgMgv1) and FgMgv1 proteins were detected using phospho-p44/42 MAP kinase antibody and the Mpk1 kinase antibody, respectively. The monoclonal anti-GAPDH antibody was used as a reference.

In order to investigate the possible role of *FgRad50* in maintaining cell wall integrity (CWI), we examined the sensitivity of ΔFgRad50 cells to the cell membrane-damaging agent SDS and the cell wall-damaging agents CR and CFW. WT strain PH-1 and the ΔFgRad50 and ΔFgRad50-C mutants were incubated on PDA medium containing 0.02% SDS, 0.2 g/L CR or 0.1 mg/mL CFW. Compared to WT PH-1 and complemented ΔFgRad50-C strains, ΔFgRad50 displayed increased tolerance to SDS, CR and CFW (Figures 4A,B).

To further confirm the involvement of *FgRad50* in the regulation of CWI, we determined the phosphorylation level of FgMgv1, a protein involved in CWI in *F. graminearum* (Xu et al., 1998; Hou et al., 2002). As shown in Figure 4C, the protein abundance of FgMgv1 was not affected by deletion of *FgRad50*. However, the phosphorylation level of FgMgv1 in ΔFgRad50 was higher than that in PH-1 and ΔFgRad50-C strains, consistent with increased tolerance of ΔFgRad50 to cell wall-damaging agents. These results indicate that *FgRad50* is associated with the CWI pathway in *F. graminearum*. A previous study reported that the phosphatase FgMsg5 functions as a negative regulator of the CWI pathway, which dephosphorylates FgMgv1 in *F. graminearum* (Yu et al., 2014). Therefore, we quantified the expression of *FgMSG5* in ΔFgRad50. However, the gene expression level of *FgMSG5* was not significant difference in *FgRad50* deletion mutant and WT PH-1 (data not shown).

### *FgRad50* Is Involved in DNA Damage Responses

**FIGURE 5:**
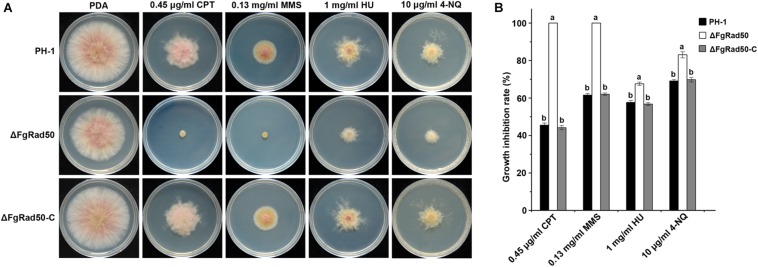
Involvement of FgRad50 in regulating sensitivity to DNA-damaging agents. **(A)** PH-1, ΔFgRad50 and ΔFgRad50-C inoculated on PDA with or without DNA-damaging agent camptothecin (CPT), methyl methanesulfonate (MMS), hydroxyurea (HU), and 4-nitroquinoline (4-NQ) at various concentrations. **(B)** Statistical analysis of the growth inhibition rate of PH-1, ΔFgRad50 and ΔFgRad50-C in the presence of the above compounds. Bars in each column denote standard deviations of three experiments. Values on the bars followed by the same letter indicate no significant difference at *P* = 0.05.

To explore the role of FgRad50 in DNA damage responses, PH-1, ΔFgRad50 and ΔFgRad50-C strains were cultured on PDA medium containing four chemical reagents, namely 0.45 μg/mL CPT, 0.13 mg/mL MMS, 1 mg/mL HU, or 10 μg/mL 4-NQ that cause different types of DNA damage. The ΔFgRad50 mutant exhibited significantly increased sensitivity to all DNA-damaging agents, and was completely inhibited (100%) by 0.45 μg/mL CPT and 0.13 mg/mL MMS, compared with PH-1 and ΔFgRad50-C strains (Figures 5A,B).

**FIGURE 6:**
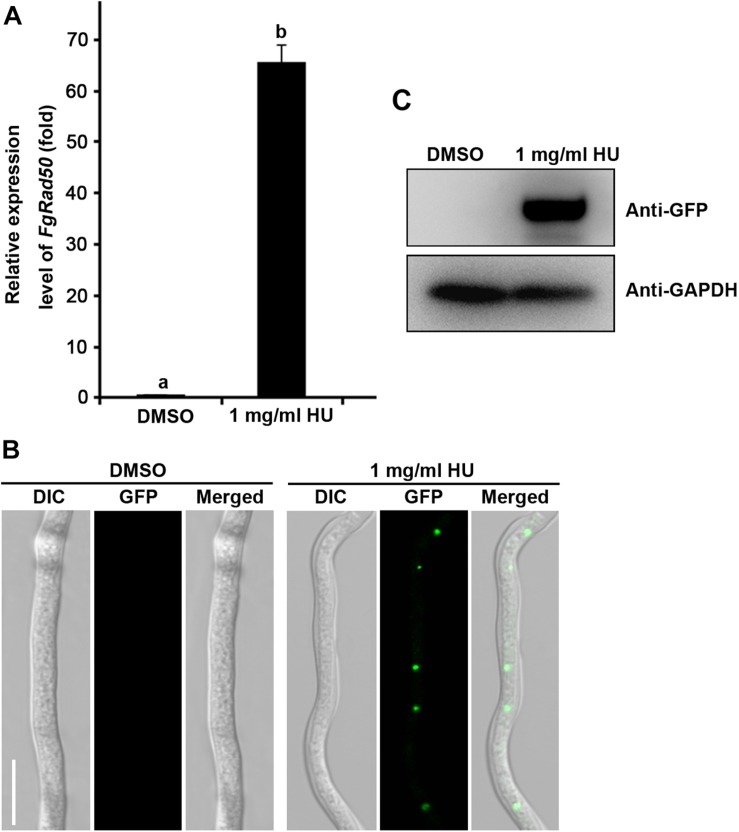
Expression pattern of FgRad50 in response to hydroxyurea (HU) induction. **(A)** The relative transcription level of *FgRad50* in WT PH-1 treated with 1 mg/mL HU is the relative amount of mRNA transcript of the gene in PH-1 without HU treatment (i.e., the solvent DMSO control). **(B)** Subcellular localization of the FgRad50-GFP transformant (ΔFgRad50-C) after HU induction. The FgRad50-GFP strain was inoculated in potato dextrose broth (PDB) and cultured at 25°C for 36 h with shaking, then treated with or without 1 mg/mL HU for 30 min, and imaged. DIC, differential interference contrast. Bar = 10 μm. **(C)** FgRad50 protein content was dramatically increased after 1 mg/mL HU treatment for 30 min determined by Western blotting with monoclonal anti-GFP antibody. The protein samples were also detected with monoclonal anti-GAPDH antibody as a reference.

To further confirm whether the expression of *FgRad50* was induced by the DNA-damaging agent, we determined *FgRad50* expression levels in WT PH-1 after treatment with 1 mg/mL hydroxyurea (HU) for 30 min. As shown in Figure 6A, the relative expression level of *FgRad50* was increased 64-fold. In addition, to examine the effect of HU on subcellular localization of FgRad50, the *FgRad50* gene fused with GFP to generate the ΔFgRad50-C strains, and this was treated with 1 mg/mL HU for 30 min. Microscopic examination and immunoblotting showed a strong GFP fluorescence signal in mycelia after treatment with HU, but no such signal when treated with the solvent dimethyl sulphoxide (DMSO; Figures 6B,C), indicating that expression of *FgRad50* is induced by the DNA-damaging agent. These results suggest that *FgRad50* plays a very important role in DNA damage responses.

### *FgRad50* Is Required for Full Virulence and DON Biosynthesis

**FIGURE 7:**
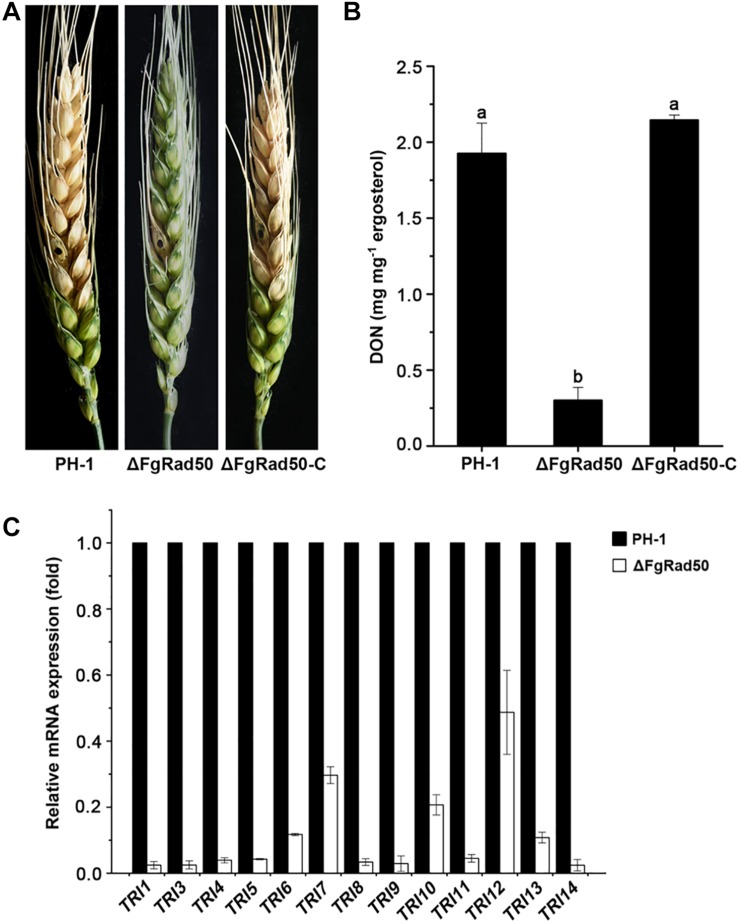
Impact of *FgRad50* deletion on virulence and DON biosynthesis in *F. graminearum*. **(A)** Disease symptoms on wheat heads caused byΔFgRad50 and ΔFgRad50-C. Wheat heads were point-inoculated with a conidial suspension of each strain, and infected wheat heads were observed 15 days after inoculation. **(B)** Levels of DON produced by each strain in infected spikelets collected from inoculated wheat heads. Error bars denote standard errors of three replicate experiments. Means of bars followed by the same letter are not significantly different at *p* = 0.05. **(C)** Relative transcription levels of 13 *TRI* genes in WT PH-1 and ΔFgRad50. For each gene, the expression level in PH-1 was arbitrarily set as 1. Bars denote standard errors from three repeated experiments.

Pathogenicity assays showed that disruption of *FgRad50* caused a dramatic reduction in the virulence of *F. graminearum*. After incubation for 15 days, WT PH-1 and ΔFgRad50-C strains exhibited severe head blight symptoms within and near inoculated spikelets of flowering wheat heads, but the ΔFgRad50 mutant displayed no such scab symptoms beyond the inoculation sites under the same conditions (Figure 7A).

Previous studies showed that DON is a key virulence factor in *F. graminearum* (Seong et al., 2009). We therefore assessed DON production in infected wheat kernels. The amount of DON produced by ΔFgRad50 was significantly lower than that produced by PH-1 and complemented strains (Figure 7B). To further confirm these results, we measured the expression levels of trichothecene (TRI) synthase genes encoding proteins responsible for DON biosynthesis by quantitative real-time PCR (qRT-PCR). As expected, expression levels of 13 *TRI* genes (*TRI1*, *TRI3*, *TRI4*, *TRI5*, *TRI6*, *TRI7*, *TRI8*, *TRI9*, *TRI10*, *TRI11*, *TRI12*, *TRI13*, and TRI14) were decreased in the ΔFgRad50 mutant compared with those in WT PH-1 cells (Figure 7C). These results indicate that FgRad50 modulates DON biosynthesis by regulating the expression of *TRI* genes in *F. graminearum*.

## Discussion

Rad50 is involved in repair of DSBs, meiotic recombination, radiation-inducible mitotic recombination and telomere maintenance in yeast (Malone et al., 1990; Merino et al., 2000; Trujillo et al., 2003; Deshpande et al., 2014). In this study, we characterized a putative homolog of *S. cerevisiae* Rad50 in *F. graminearum*. FgRad50 restored sensitivity to CPT in the yeast ΔRad50 deletion mutant (Figure 1B), indicating that this protein shares a conserved function associated with DSB repair in *F. graminearum*, and possibly in other filamentous fungi. In addition, deletion of *FgRad50* led to reduced vegetative growth, conidiation, DON production and virulence in wheat heads.

The function of Rad50 has been widely studied in yeast and humans (Gatei et al., 2011; Seifert et al., 2016), but not filamentous fungi. The present study showed that the ΔFgRad50 mutant of *F. graminearum* exhibited severe defects in vegetative growth (Figure 2A), but in yeast, the Rad50 gene is not essential for vegetative growth (Kupiec, 1986). The reason for this apparent discrepancy requires further research. In addition, the ΔFgRad50 mutant displayed diminished conidiation (Figure 2B) and perithecia formation (Figure 3A) compared with the PH-1 strain, and microscopic examination revealed that the ΔFgRad50 mutant was unable to form asci (Figure 3B). These results indicate that *FgRad50* is important for sexual and asexual developmental processes in *F. graminearum*.

The phosphorylation level of FgMgv1 has been shown to be associated with resistance to cell wall stress (Wang et al., 2011; Yun et al., 2014). In this study, we found that the mutant ΔFgRad50 had a higher level of FgMgv1 phosphorylation in comparison with the WT PH-1 (Figure 4C). However, there was not significant difference of the transcript level of *FgMSG5* in ΔFgRad50 and wild type, which encodes a phosphatase that dephosphorylates FgMgv1. These results indicate that FgRad50 plays a key role in the response of *F. graminearum* to cell wall stress via negative regulation of FgMgv1 phosphorylation, but not positively regulates transcription of *FgMSG5*. Thus, further studies are still needed to explore the relationship between FgRad50 and FgMgv1 in *F. graminearum.* Additionally, the mutant was relatively sensitive to DNA-damaging agents CPT, MMS, HU and 4-NQ (Figures 5A,B). The sensitivity of ΔFgRad50 to these drugs suggests that ΔFgRad50 functions in a chromosome transmission checkpoint response and/or supports chromosome transmission, similar to that reported in a yeast model (Williams et al., 2011; Deshpande et al., 2014).

In addition to its involvement in regulating mycelial growth, conidiation and DNA-damaging agent sensitivity, *FgRad50* is also required for the virulence of *F. graminearum*. The reduced virulence of ΔFgRad50 may result from two defects in the mutant. Firstly, expression levels of 13 *TRI* genes encoding proteins responsible for DON biosynthesis were down-regulated, consequently decreasing DON production by ΔFgRad50 in infected wheat kernels. Interestingly, DON plays an important role in the spread of FHB within a flowering spike (Seong et al., 2009). Second, the mutant ΔFgRad50 grew significantly slower than the WT strain on CM medium (Figure 2). Thus, its reduced growth rate may also contribute to the reduced virulence of the mutant. Although Rad50 is present in wheat and human, multiple sequence alignments illustrated that FgRad50 is divergent enough from orthologs in wheat and human. Therefore, new drugs can be designed exclusively targetting the FgRad50 and not have adverse effects on the crops and human as the novel fungicide JS399-19 species specifically targets the myosin I of *F. graminearum* (Zhang et al., 2015).

In summary, we identified *FgRad50* that is involved in DNA damage responses in *F. graminearum*, as well as vegetative growth, conidiation and virulence. To our knowledge, this is the first report about the functions of Rad50 in phytopathogenic fungi, which appear to differ from those reportedly previously in yeast.

## Data Availability Statement

All datasets generated for this study are included in the article/Supplementary  Material.

## Author Contributions

CZ and LC conceived and designed the experiments. CZ, XR, XW, and QW performed the experiments. CZ wrote the manuscript. LC and KD supervised the whole the work and the revision of the manuscript.

## Conflict of Interest

The authors declare that the research was conducted in the absence of any commercial or financial relationships that could be construed as a potential conflict of interest.

## Footnotes

1 https://fungidb.org/fungidb/

## Supplementary Material

FIGURE S1 Generation and confirmation of the deletion mutant ΔFgRad50 and complemented strain ΔFgRad50-C. **(A)** Schematic representation of the *FgRad50* disruption strategy. *FgRad50* and the hygromycin resistance cassette (*HPH*) are denoted by open and gray boxes, respectively. **(B)** A 841 bp upstream fragment of *FgRad50* was used as a probe in Southern hybridization analysis. Genomic DNA from PH-1, ΔFgRad50 and ΔFgRad50-C strains was digested with *Eco*RI restriction enzyme. The wild type strain had an expected hybridizing band of 3459-bp, while this band was replaced by a 6509-bp fragment in ΔFgRad50. Additionally, the southern hybridization pattern (the top and bottom band) of ΔFgRad50-C showed that a single copy of the *pYF11-FgRad50- GFP-Gen* fusion vector was re-introduced as random integration into the genome of ΔFgRad50 and was digested into two parts by *Eco*RI.

FIGURE S2 Comparison of FgRad50 with homologs from *Homo sapiens* (HsRad50) and *Triticum turgidum* (TtRad50). Alignments of amino acid sequences of FgRad50 among homologs from HsRad50 and TtRad50. Genedoc software was used to highlight identical (black shading) or similar (gray shading) amino acids.

TABLE S1 PCR primers used in this study.
