# Supplementary figures and images for: FgRad50 Regulates Fungal Development, Pathogenicity, Cell Wall Integrity and the DNA Damage Response in Fusarium graminearum
Source: Front Microbiol. 2020 Jan 9;10:2970. doi: 10.3389/fmicb.2019.02970 (PMC6962240; doi:10.3389/fmicb.2019.02970)

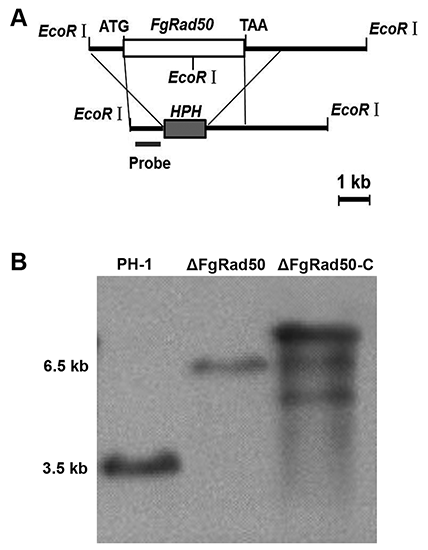

Supplement: FIGURE S1 — Generation and confirmation of the deletion mutant ΔFgRad50 and complemented strain ΔFgRad50-C. (A) Schematic representation of the FgRad50 disruption strategy. FgRad50 and the hygromycin resistance cassette (HPH) are denoted by open and gray boxes, respectively. (B) A 841 bp upstream fragment of FgRad50 was used as a probe in Southern hybridization analysis. Genomic DNA from PH-1, ΔFgRad50 and ΔFgRad50-C strains was digested with EcoRI restriction enzyme. The wild type strain had an expected hybridizing band of 3459-bp, while this band was replaced by a 6509-bp fragment in ΔFgRad50. Additionally, the southern hybridization pattern (the top and bottom band) of ΔFgRad50-C showed that a single copy of the pYF11-FgRad50- GFP-Gen fusion vector was re-introduced as random integration into the genome of ΔFgRad50 and was digested into two parts by EcoRI. [file Image_1.TIF]

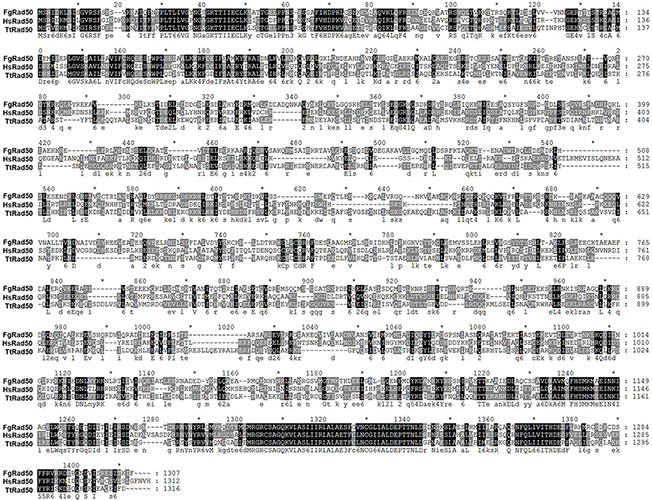

Supplement: FIGURE S2 — Comparison of FgRad50 with homologs from Homo sapiens (HsRad50) and Triticum turgidum (TtRad50). Alignments of amino acid sequences of FgRad50 among homologs from HsRad50 and TtRad50. Genedoc software was used to highlight identical (black shading) or similar (gray shading) amino acids. [file Image_2.TIF]
