# Supplementary material for: FgRad50 Regulates Fungal Development, Pathogenicity, Cell Wall Integrity and the DNA Damage Response in Fusarium graminearum
Source: Front Microbiol. 2020 Jan 9;10:2970. doi: 10.3389/fmicb.2019.02970 (PMC6962240; doi:10.3389/fmicb.2019.02970)
Supplement: TABLE S1 — PCR primers used in this study. [file Table_1.DOC]

**Table S1** Oligonucleotide primers used in this study

| Code | Primer | Sequence (5’-3’) | Relevant characteristics |
| --- | --- | --- | --- |
| 1 | A1 | GGTGTCTCATTCAAGAGTCAAG | A pair of PCR primers for amplification of the upstream sequence of *FgRad50* for construction of the gene deletion |
| 2 | A2 | CAAAATAGGCATTGATGTGTTGACCTCCGGTGATGACTACCACATTAA |
|  |  |  |  |
| 3 | A3 | CTCGTCCGAGGGCAAAGGAATAGAGTAGTCGGGCATGCAAGTTGAGTA | A pair of PCR primers for amplification of the downstream sequence of *FgRad50* for construction of the gene deletion |
| 4 | A4 | CACTTCCTATCCAGACGTAAC |
|  |  |  |  |
| 5 | A5 | TGATGGGAATACGTACCGTG | A pair of PCR primers for identification of *FgRad50* deletion mutants |
| 6 | A6 | TGGAGAAATAATTCCGTTGC |
|  |  |  |  |
| 7 | A7 | ACTCACTATAGGGCGAATTGGGTACTCAAATTGGTTGTCGAGACATGCATATAATGT | A pair of PCR primers to amplify *FgRad50* fragments used for construction of the FgRad50-GFP vector under its own promoter |
| 8 | A8 | CACCACCCCGGTGAACAGCTCCTCGCCCTTGCTCACGAATATCTTTGTAATACTTTC |
|  |  |  |  |
| 9 | A9 | GGAGGTCAACACATCAATGCCTATT | PCR primers for amplification of hygromycin resistance gene (*HPH*) |
| 10 | A10 | CTACTCTATTCCTTTGCCCT |
|  |  |  |  |
| 11 | A11 | CTTGGTACCGAGCTCGGATCCATGTCTAGAATCGATAAGCTTT | PCR primers for amplification of full cDNA sequence of the *FgRad50* gene for yeast complementation experiment |
| 12 | A12 | CCCTCTAGATGCATGCTCGAGTTAGAATATCTTTGTAATAC |
|  |  |  |  |
| 13 | A13 | TCACTACAATCGTCTTCTGGC | Quantitative real-time PCR primers for analysis of the gene *FgRad50* expression level |
| 14 | A14 | CAATGTCGGTTCCTTGGTAGG |
|  |  |  |  |
| 15 | RT-Tri1-F | GATGTTCCTTCTCGACAGCGT | Quantitative real-time PCR primers for analysis of the gene *TRI1* expression level |
| 16 | RT-Tri1-R | CACTGGTCGAAGATAGCTGG |
|  |  |  |  |
| 17 | RT-Tri3-F | TGTTACGATCAATGGCTTGG | Quantitative real-time PCR primers for analysis of the gene *TRI3* expression level |
| 18 | RT-Tri3-R | TCCTCGTTGTAGTTTGCATCA |
|  |  |  |  |
| 19 | RT-Tri4-F | ACGTGTGGCTACTCAGGAGAA | Quantitative real-time PCR primers for analysis of the gene *TRI4* expression level |
| 20 | RT-Tri4-R | TGGAATTGCCTTGGGGTA |
|  |  |  |  |
| 21 | RT-Tri5-F | ATGGCGGATCTATCTATTCAC | Quantitative real-time PCR primers for analysis of the gene *TRI5* expression level |
| 22 | RT-Tri5-R | CCATTCATACGACGAAGGAAT |
|  |  |  |  |
| 23 | RT-Tri6-F | AAATGCCCATTCCCTAGTTG | Quantitative real-time PCR primers for analysis of the gene *TRI6* expression level |
| 24 | RT-Tri6-R | ATCTCGCATGTTATCCACCCT |
|  |  |  |  |
| 25 | RT-Tri7-F | TACCGTCGTCTTCAAAACCA | Quantitative real-time PCR primers for analysis of the gene *TRI7* expression level |
| 26 | RT-Tri7-R | ACGCCAATGGTGTTCACAAA |
|  |  |  |  |
| 27 | RT-Tri8-F | ATATAACGGTACCCCCAGATG | Quantitative real-time PCR primers for analysis of the gene *TRI8* expression level |
| 28 | RT-Tri8-R | TGTTTGTAGGACACTTCCGGT |
|  |  |  |  |
| 29 | RT-Tri9-F | CCGCTAAACTGATCGACTCAT | Quantitative real-time PCR primers for analysis of the gene *TRI9* expression level |
| 30 | RT-Tri9-R | CCCATATGGTAGCGCATAAA |
|  |  |  |  |
| 31 | RT-Tri10-F | TCCCAACCTTTCAGAGGTTCA | Quantitative real-time PCR primers for analysis of the gene *TRI10* expression level |
| 32 | RT-Tri10-R | TGATCCGTCAAGTCTTCCCAT |
|  |  |  |  |
| 33 | RT-Tri11-F | TGAGAACGACATGTGGGCAAT | Quantitative real-time PCR primers for analysis of the gene *TRI11* expression level |
| 34 | RT-Tri11-R | AGGCTTGTTCCATGCAAGAT |
|  |  |  |  |
| 35 | RT-Tri12-F | ACGAACAGCACTGCTACGGT | Quantitative real-time PCR primers for analysis of the gene *TRI2* expression level |
| 36 | RT-Tri12-R | TTCCTGCTTGTGACTCCAAT |
|  |  |  |  |
| 37 | RT-Tri13-F | ACGCAGATCCTGGGATATCA | Quantitative real-time PCR primers for analysis of the gene *TRI13* expression level |
| 38 | RT-Tri13-R | CAGCCCAGTATTTTGCCAAA |
|  |  |  |  |
| 39 | RT-Tri14-F | AACTCCCGTTGTGATCAAGCA | Quantitative real-time PCR primers for analysis of the gene *TRI14* expression level |
| 40 | RT-Tri14-R | AACAGTAATGTTGGCACCGT |
|  |  |  |  |
| 41 | RT-Tri101-F | CATCACGCCTTCGTTCAGAAC | Quantitative real-time PCR primers for analysis of the gene *TRI101* expression level |
| 42 | RT-Tri101-R | CATCAAGCTCTCAACAGGCT |
|  |  |  |  |
| 43 | RT-actin-F | ATCCACGTCACCACTTTCAA | Quantitative real-time PCR primers for analysis of the reference gene *actin* expression level |
| 44 | RT-actin-R | TGCCTTGAGATCCACATTTG |
